# Long non-coding RNA-H19 antagonism protects against renal fibrosis

**DOI:** 10.18632/oncotarget.10444

**Published:** 2016-07-06

**Authors:** Hong Xie, Jing-Dong Xue, Feng Chao, Yan-Feng Jin, Qiang Fu

**Affiliations:** ^1^ Department of Urology, Shanghai Jiao Tong University Affiliated Sixth People's Hospital, Shanghai, China

**Keywords:** long non-coding RNA, renal fibrosis, microRNA, unilateral ureteral obstruction

## Abstract

Although long non-coding RNAs (lncRNAs) are important players in the initiation and progression of many pathological processes, the role of lncRNAs in renal fibrosis still remains unclear. We showed that lncRNA-H19 expression was significantly up-regulated in TGF-β2-induced HK-2 cell fibrosis and unilateral ureteral obstruction (UUO)-induced renal fibrosis *in vivo*. H19 knockdown significantly attenuated renal fibrosis *in vitro* and *in vivo.* LncRNA-H19, miR-17, and fibronectin constituted to a regulatory network involved in renal fibrosis. We also detected up-regulated H19 expression and down-regulated miR-17 expression in the early and advanced animal models of renal fibrosis. This study indicates that H19 up-regulation contributes to renal fibrosis. H19 inhibition might represent a novel anti-fibrotic treatment in renal diseases.

## INTRODUCTION

Renal fibrosis is usually the final outcome of many renal diseases [1]. Many cellular and molecular events occur in renal fibrosis, such as activation of interstitial fibroblasts, phenotypic conversion of tubular epithelial and endothelial cells, extracellular matrix (ECM) overproduction, and microvascular dysfunction [2–4]. Excessive renal fibrosis would result in scar formation, and ultimately causes renal failure [5]. Thus, further understanding of the underlying mechanism of renal fibrosis would provide new opportunities for therapeutic development.

Non-coding RNAs have been reported to play important roles in renal diseases [6]. Identification of renal disease-related miRNAs was previously recognized as a promising method for renal disease therapy [7]. However, one miRNA can simultaneously regulate many target genes. The off-target effects hinder the application of miRNA therapy [8, 9]. LncRNAs are non-coding transcripts greater than 200 nucleotides [10]. Previous studies have revealed that mutation and dysregulation of lncRNAs are tightly associated with human diseases ranging from neurodegeneration to cancer [11, 12]. Some experiments have revealed that aberrant lncRNA expression occurs in renal development, renal cell carcinoma, and renal inflammation [13–15].

H19 is a 3 kb non-coding RNA expressed in nucleus and cytoplasm. H19 is activated in embryonic cells and highly expressed in embryogenesis. H19 expression is significantly decreased after birth, but is significantly increased in diseased condition [16–18]. H19 has been reported to play an important role in renal development [14, 19]. However, the role of H19 in renal diseases still remains unclear. In this study, we investigate the role of H19 in renal fibrosis using TGF-β2-induced renal cell model and mouse model of renal fibrosis.

## RESULTS

### LncRNA-H19 expression is up-regulated in renal fibrosis

**Figure 1:**
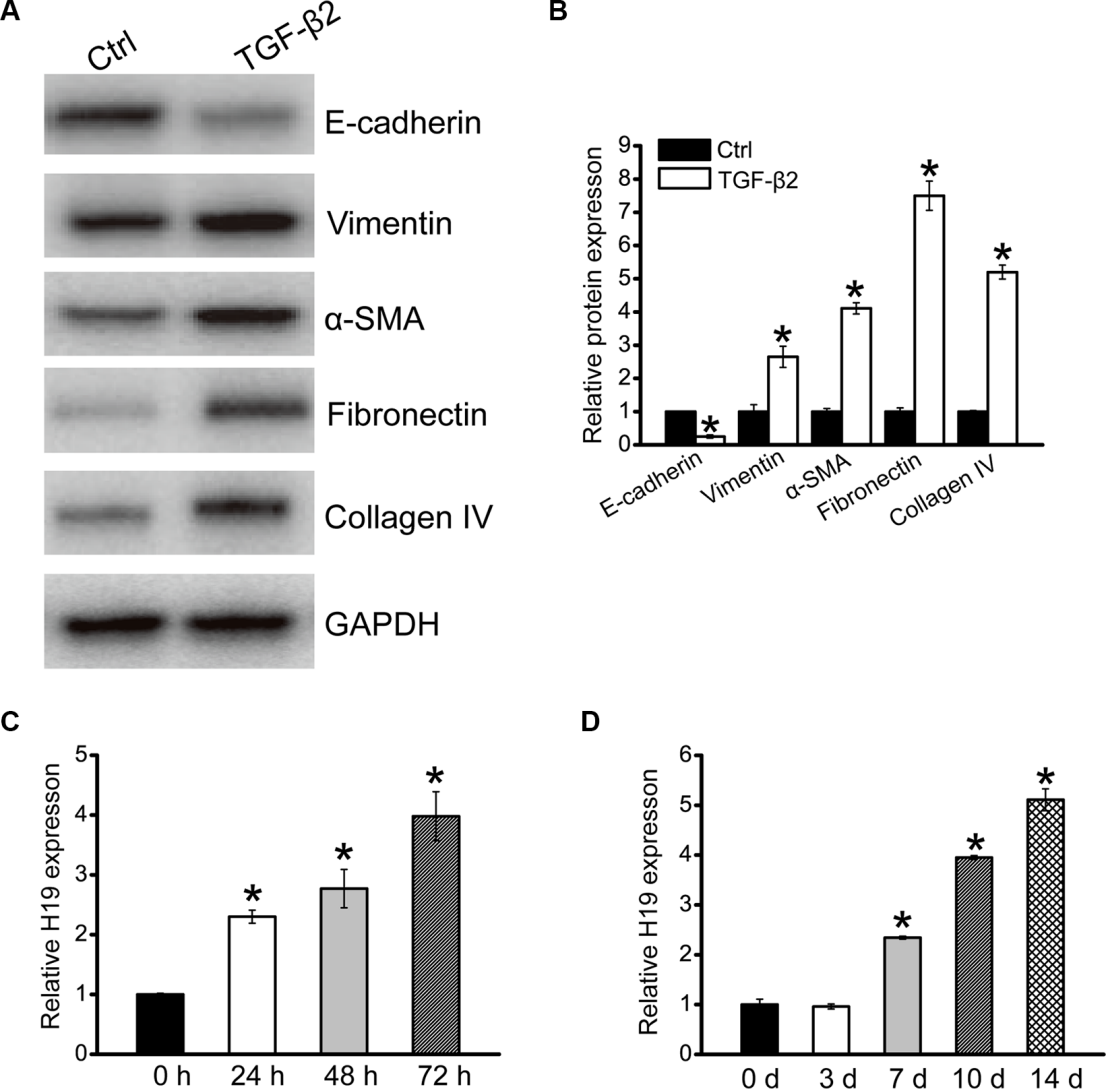
LncRNA-H19 expression is up-regulated in renal fibrosis (**A**, **B**) HK-2 cells were cultured with or without TGF-β2 (10 ng/ml) for 3 days. After TGF-β2 treatment, western blots were conducted to detected E-cadherin, vimentin, α-SMA, fibronectin, and collagen IV expression. GAPDH was detected as the internal control. Protein expression was determined as the ratio of densitometric value compared to GAPDH expression. Representative immunoblots were shown along with the quantitative data (*n* = 4; *P < 0.05 compared with control). (**C**) HK-2 cells were cultured in the presence of TGF-β2 (10 ng/ml) for the indicated time periods. qRT-PCRs were conducted to detect the expression of H19. GAPDH was detected as the internal control (*n* = 4; *P < 0.05). (**D**) qRT-PCRs were conducted to detect renal H19 levels in UUO-induced obstructive nephropathy model at the indicated time points. GAPDH was detected as the internal control (*n* = 4; *P < 0.05). All data were from three independent experiments.

HK-2 cells were exposed to TGF-β2 (10 ng/ml) for 3 days to induce fibrosis. We found that HK-2 cells displayed phenotypic transition of epithelial-mesenchymal transition (EMT), which is an important feature of fibrosis, characterized by down-regulated levels of epithelial protein marker (E-cadherin), up-regulated levels of mesenchymal protein marker (vimentin and α-SMA), and ECM proteins (fibronectin and collagen IV) (Figure 1A and 1B). TGF-β2 treatment also led to a significant increase in H19 expression in time-dependant manner *in vitro* (Figure 1C). Moreover, we determined H19 expression in a renal fibrosis mouse model of UUO nephropathy. H19 expression was significantly up-regulated in UUO kidney as early as 7 days after operation (Figure 1D). Collectively, these results indicate that H19 expression is significantly up-regulated during renal fibrosis *in vivo* and *in vitro*.

### LncRNA-H19 knockdown inhibits renal fibrosis *in vivo*

We then determine the role of H19 in the development of renal fibrosis *in vivo*. Viral shRNA injection did not lead to a significant immune response. Compared with PBS or scrambled shRNA-injected mice, IL-6 and monocyte chemoattractant protein 1 (MCP-1) levels in the serum of C57BL/6 mice did not obviously change (Supplementary Figure S1A). H19 expression was reduced by H19 shRNA injection, but not by or PBS injection (Supplementary Figure S1B). Moreover, H19 shRNA injection specifically reduced the expression of H19, but not other lncRNAs, including MALAT1, XIST, MEG3, and GAS5 (Supplementary Figure S1C).

**Figure 2:**
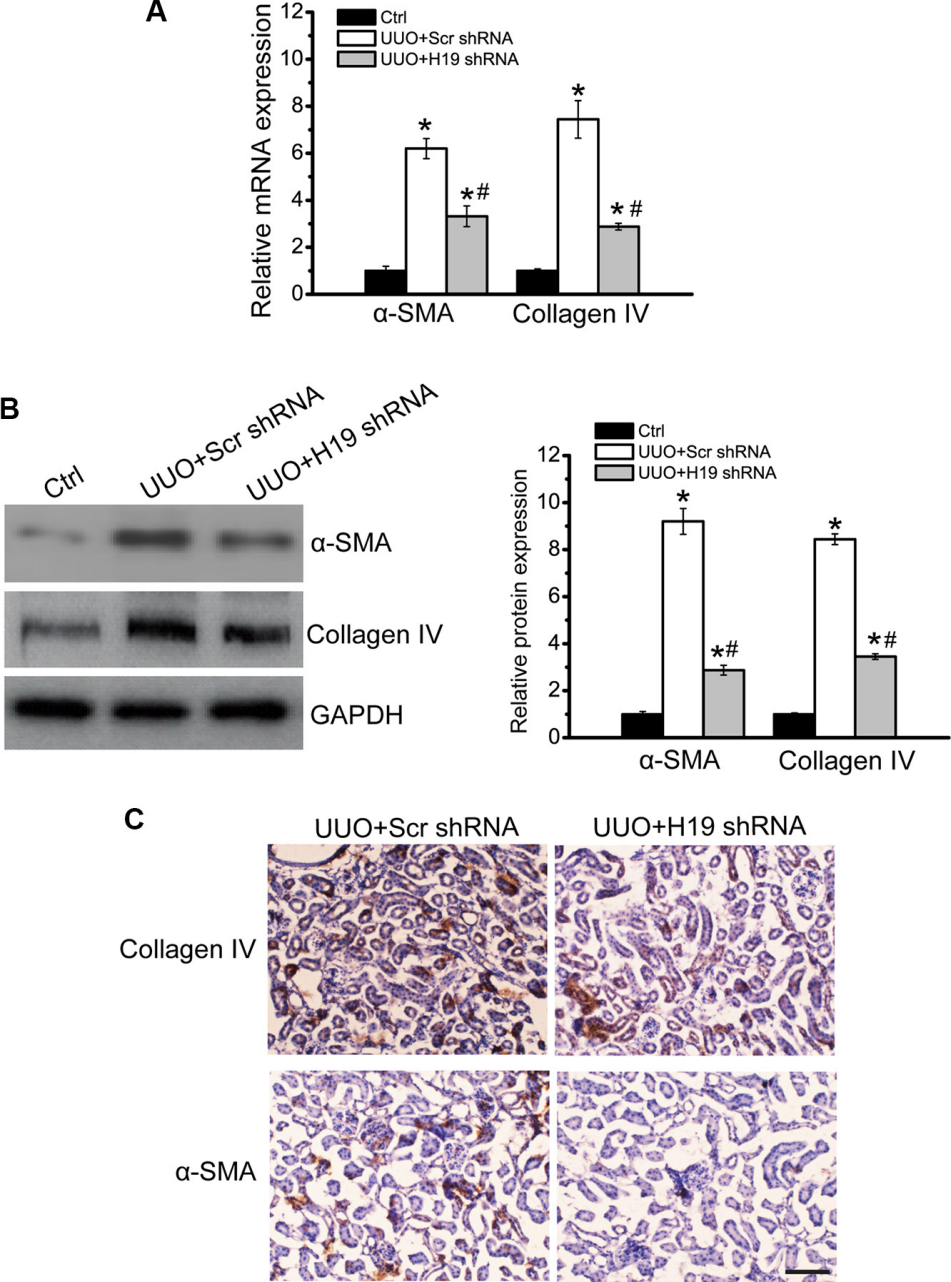
LncRNA-H19 knockdown inhibits renal fibrosis *in vivo* (**A**, **B**) Age- and sex-matched H19 knockdown and wild-type C57BL/6 mice (four-month old; male) were subjected to UUO maneuver and killed after 14-day treatment. qRT-PCRs (A) and western blots (B) were conducted to detect the levels of collagen IV and α-SMA. GAPDH was determined as the internal control. Representative immunoblots were shown along with the quantitative data showing mean ± S.E.M. from four separate blots (*n* = 4; *P < 0.05 compared with control). (**C**) Immunohistochemistry analysis of the accumulation of α-SMA and deposition of collagen IV were conducted in an established mouse model of UUO nephropathy at day 14. Scale bar: 20 μm (*n* = 5 animals per group). All data were from three independent experiments.

We next investigated whether H19 knockdown affected the development of renal fibrosis. Age- and sex-matched H19 knockdown and wild-type mice were subjected to UUO maneuver and killed after 14-day treatment. UUO kidney displayed obvious renal fibrosis as shown by increased expression of α-SMA and collagen IV, whereas H19 knockdown reduced the up-regulation of α-SMA and collagen IV expression at gene and protein levels (Figure 2A and 2B). Immunohistochemistry assay also revealed that H19 knockdown significantly decreased the accumulation of α-SMA and deposition of collagen IV (Figure 2C). These results suggest that H19 knockdown inhibits renal fibrosis *in vivo*.

### LncRNA-H19 knockdown affects renal cell function and decreases renal fibrosis *in vitro*

**Figure 3:**
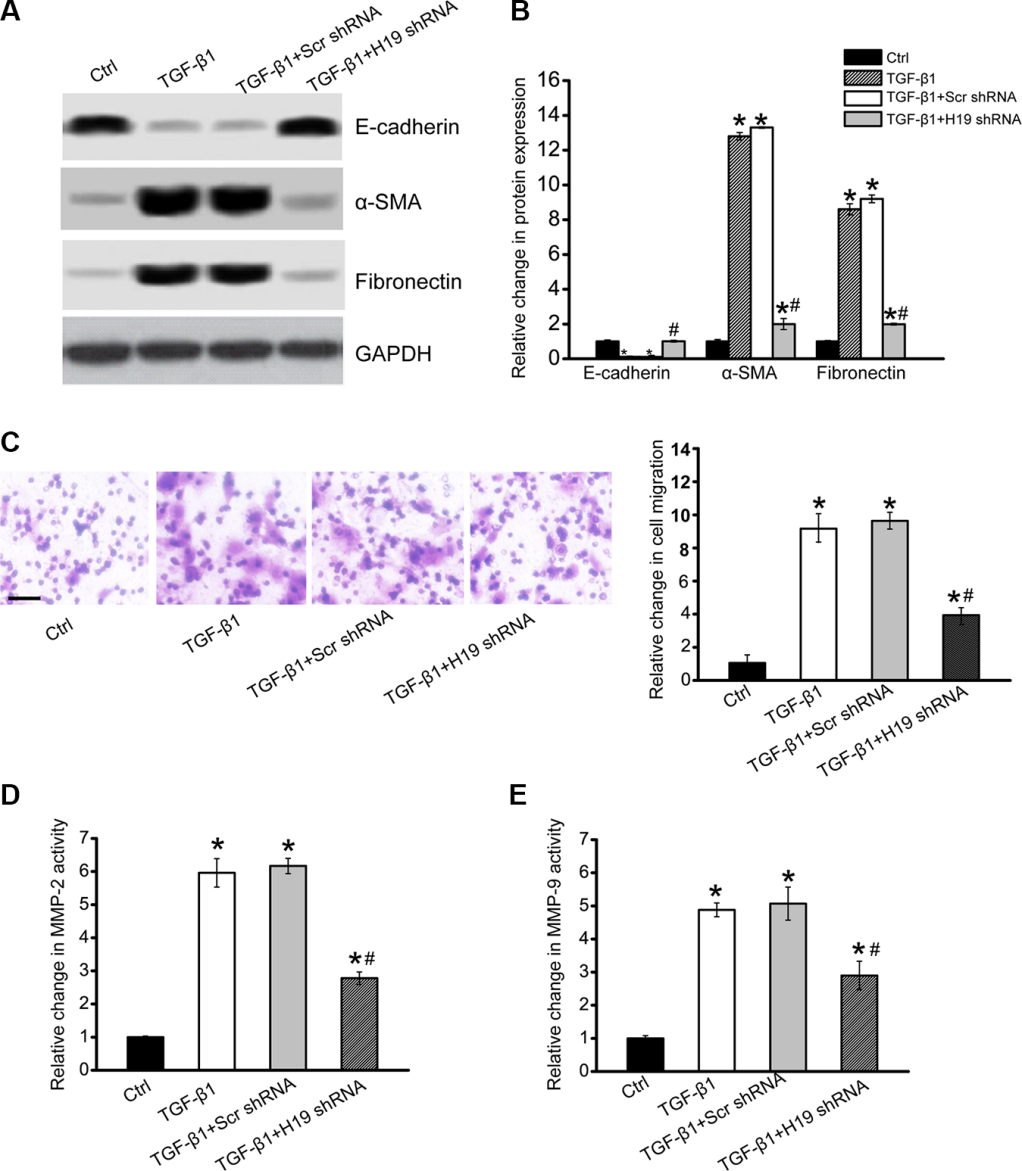
LncRNA-H19 knockdown affects renal cell function and inhibits renal fibrosis *in vitro* (**A**, **B**) HK-2 cells were transfected with H19 siRNA, scrambled siRNA (Scr), or left untreated, and then cultured in the presence of TGF-β2 (10 ng/ml) for 48 h. The untreated group was taken as the control group (Ctrl). Western blots were conducted to detect E-cadherin, α-SMA, and fibronectin. GAPDH was detected as the internal control. Protein expression was determined as the ratio of densitometric value compared to GAPDH expression. Representative immunoblots were shown along with the quantitative data showing mean ± S.E.M. from four separate blots (*n* = 4; *P < 0.05 compared with control). (**C**) Transwell assay and quantitative analysis was performed to detect HK-2 cell migration (*n* = 4). Scale bar, 50 μm. (**D**, **E)** ELISA assays were conducted to detect the activity of MMP-2 and MMP-9 in the culture medium of HK-2 cells after the required treatment (*n* = 4; *P < 0.05 versus Ctrl group; ^#^*P* < 0.05 TGF-β2 group versus TGF-β2+H19 siRNA group). All data were from three independent experiments.

We next investigated the functional significance of H19 knockdown *in vitro*. H19 siRNA transfection significantly down-regulated H19 expression in HK-2 cells (Supplementary Figure S2). EMT is recognized as a mechanism by which injured renal tubular cells transform into mesenchymal cells that contribute to renal fibrosis [20]. In TGF-β2-induced cell fibrosis, there were significant changes in HK-2 cell phenotypes, shown as decreased E-cadherin expression and increased expression of α-SMA and fibronectin. H19 knockdown partially reversed these changes, decreasing the loss of E-cadherin expression and reducing α-SMA and fibronectin expression (Figure 3A and 3B). Renal cells lost their epithelial phenotype and acquisition of myofibroblastic phenotype during EMT process, such as increased motility, invasiveness, and extracellular protein synthesis. Transwell assays showed that H19 knockdown partially decreased TGF-β1-induced cell migration (Figure 3C). ELISA assays showed that H19 knockdown partially reduced TGF-β2-induced increased MMP-2 and MMP-9 activity (Figure 3D and 3E). These results showed that H19 is involved in regulating renal cell function and fibrosis *in vitro*.

### LncRNA-H19 functions as miR-17 sponge in renal cells

**Figure 4:**
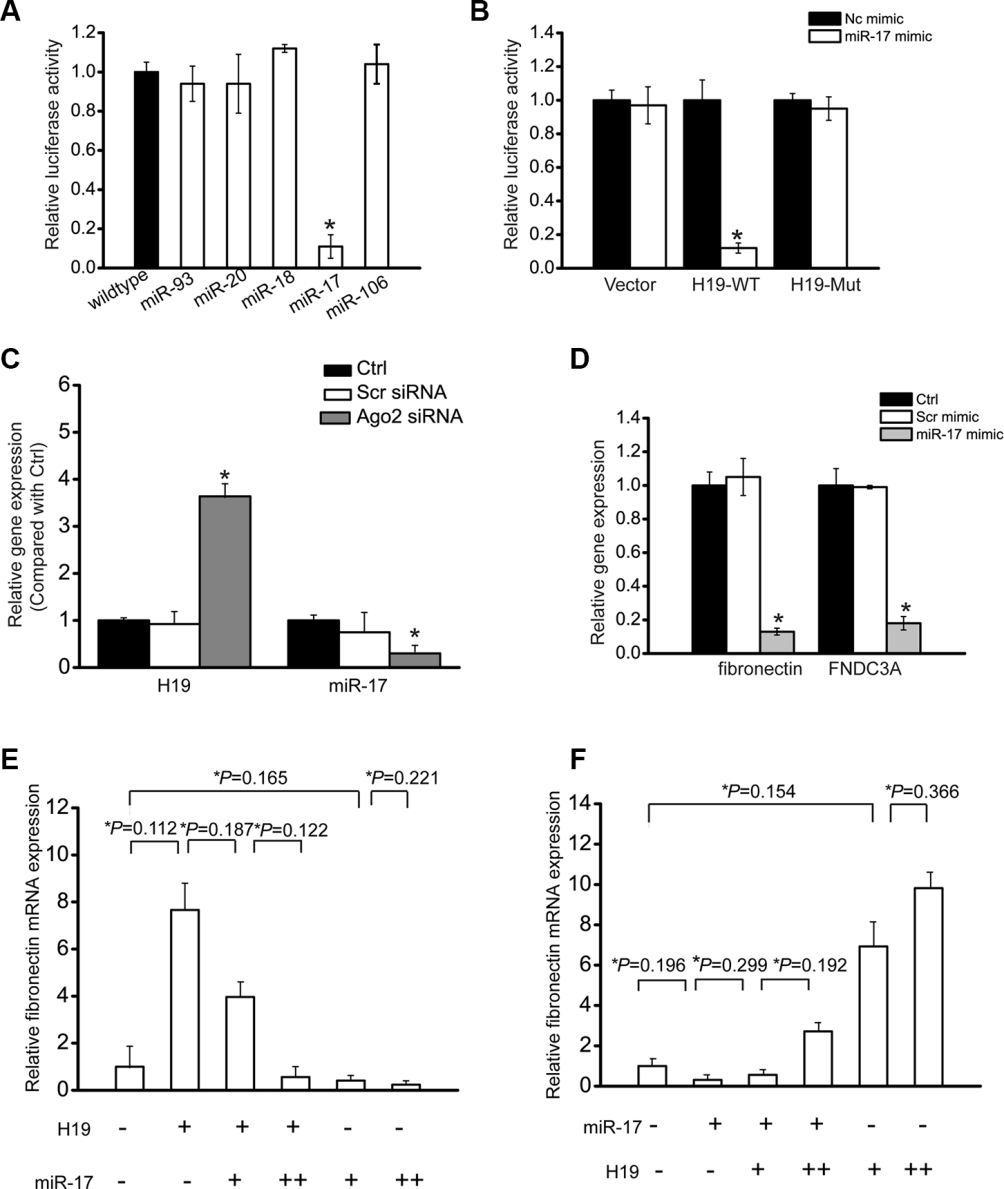
LncRNA-H19 functions as miR-17 sponge in renal cells . (**A**) HK-2 cells were co-transfected RLuc-H19-WT with different miRNA mimics. Luciferase activity was detected using the dual luciferase assay (Promega). The group only transfected with RLuc-H19-WT vector was taken as the control group. Luciferase activity was detected 48 h after transfection (*n* = 4). (**B**) RLuc-H19-WT or RLuc-H19-Mut was co-transfected with miR-17 mimic into HK-2 cells in parallel with the vector. Luciferase activity was detected 48 h after transfection. The data was shown as relative change compared with the control group (*n* = 4). (**C**) HK-2 cells were transfected with Ago2 siRNA, scrambled siRNA, or left untreated (Ctrl). miR-17 or H19 levels were detected using qRT-PCRs (*n* = 4). (**D**) HK-2 cells were transfected with miR-17 mimic, scrambled mimic, or left untreated (Ctrl). Fibronectin and FNDC3A levels were detected using qRT-PCRs (*n* = 4). (**E**, **F**) HK-2 cells were transfected with different combinations of H19 and miR-17 mimic. qRT-PCRs were conducted to detect fibronectin expression. (+) corresponds to 24 ng H19 construct or 12 ng of miR-17 mimic. (++) corresponds to 50 ng H19 construct or 25 ng of miR-17 mimic. Data was shown as mean ± S.E.M., and expressed as the relative change compared with the control group (*n* = 4). ^#^*P* < 0.05 indicated significant difference between the marked groups. All data were from three independent experiments.

LncRNAs can function as miRNA sponges to regulate the availability of miRNA for binding target mRNAs [21]. We first employed StarBase 2.0 to predict miRNA recognition elements on H19 using human and mouse genome. miR-93, miR-20, miR-18, miR-106, and miR-17 was predicated as the potential miRNA targets on H19. The activity of RLuc-H19-WT was significantly decreased by miR-17 mimic transfection (Figure 4A). We also showed that miR-17 mimic transfection significantly reduced RLuc-H19-WT activity, but did not affect RLuc-H19-Mut activity (Figure 4B). Ago2 is a key component of RNA-induced silencing complex (RISC), which is involved in the miRNA-mRNA binding. We also studied whether H19 expression is regulated by miRNAs via Ago2 knockdown. Ago2 knockdown led to a significant increase in H19 expression, whereas miR-17 stability was impaired by Ago2 knockdown (Figure 4C). In previous study, fibronectin and the fibronectin type-III domain containing 3A (FNDC3A) are shown as two mRNA targets of miR-17 [22]. We found that miR-17 mimic transfection led to a marked reduction of fibronectin and FNDC3A expression in HK-2 cells, suggesting that fibronectin and FNDC3A is the target gene of miR-17 (Figure 4D).

If H19 regulates HK-2 cell function through ceRNA mechanism, it would effectively function as a decoy. The relative concentration of H19 and miRNAs could alter mRNA expression of target genes. We gradually up-regulated miR-17 levels in the presence or absence of H19. H19 led to a significant increase in fibronectin level, and was gradually reduced when miR-17 level was increased (Figure 4E). We also gradually increased H19 amount in the presence or absence of miR-17. miR-17 led to obvious decrease in fibronectin level, whereas the decrease was gradually restored when H19 level was increased (Figure 4F).

### Increased H19 expression and decreased miR-17 expression in fibrotic renal tissue

**Figure 5:**
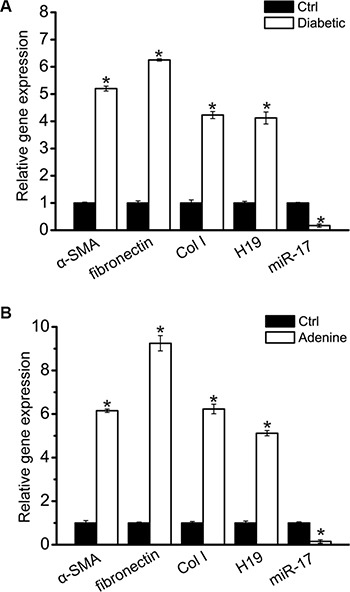
Increased H19 and decreased miR-17 in the kidney with renal fibrosis (**A**) Total mRNAs were extracted from the renal cortex of normal and 16-week diabetic C57BL/6 mice (*n* = 5 animals per group). Gene expression was determined by qRT-PCRs, including α-SMA, fibronectin, Col I, H19, and miR-17 (*P < 0.05 compared with Ctrl group). (**B**) Total mRNAs were extracted from the renal cortex from normal and adenine-fed C57BL/6 mice after 4-week treatment (*n* = 5 animals per group). Gene expression was determined by qRT-PCRs, including α-SMA, fibronectin, Col I, H19, and miR-17 (*P < 0.05 compared with Ctrl group). All data were from three independent experiments.

To further determine the expression relationship between miR-17 and H19, we also detected their expression pattern in the cortex of renal from diabetic mice. In this model, chronic hyperglycemia led to increased renal fibrosis, which is similar to the phenomena in early diabetic human renal disease. Diabetes was followed by increased expression of α-SMA, fibronectin, and Col I. H19 expression was significantly up-regulated, whereas miR-17 expression was significantly reduced (Figure 5A). We also investigated the expression pattern of miR-17 and H19 in advanced kidney disease induced by adenine. Adenine treatment led to up-regulated expression of α-SMA, fibronectin, and Col I. H19 expression was found to be significantly up-regulated, whereas miR-17 expression was significantly reduced (Figure 5B).

## DISCUSSION

Renal fibrosis is initiated and sustained by many prosclerotic factors. Among these factors, TGF-β can increase the expression of matrix proteins and induce epithelial-to-mesenchymal transition in renal cells [23]. TGF-β mainly includes three different isoforms in renal, and all of them induce renal fibrosis [24, 25]. We showed that TGF-β2 treatment significantly up-regulated lncRNA-H19 levels. H19 knockdown inhibited TGF-β2-induced renal fibrosis *in vitro* and *in vivo*. LncRNA-H19, miR-17, and fibronectin constituted to a regulatory network, and participated in renal fibrosis. Moreover, up-regulated H19 expression and down-regulated miR-17 expression was detected in the early and advanced animal models of renal fibrosis.

mRNA, microRNA, and long non-coding RNAs can communicate with each other by competing for shared miRNAs [26, 27]. We revealed that lncRNA-H19, miR-17, and fibronectin mRNA constituted a competing endogenous RNAs (ceRNA) regulatory network. This regulatory network maintained a relative balance to avoid abnormal renal fibrosis. Once lncRNA-H19 was induced in renal fibrosis, increased H19 levels could alleviate miR-17 repressive effect, and lead to increased expression of fibronectin, a target gene of miR-17. Similar regulatory mechanisms of H19 have been reported. H19 is a developmental reservoir of miR-675 that inhibits growth and Igf1r expression [28]. H19 promotes pancreatic cancer metastasis by releasing let-7-mediated inhibition on the target HMGA2-mediated EMT [29]. H19/miR-675 axis inhibits prostate cancer metastasis via affecting TGFBI expression [30]. Thus, H19 may play its role through ceRNA mechanism.

miRNAs have been reported to play critical roles in the pro-fibrotic pathways and extracellular matrix synthesis. Previous study suggests that miR-17 retards tissue growth and inhibits fibronectin expression [22]. Epigenetic modulation of miR-17~92 contributes to the development of pulmonary fibrosis [31]. We revealed a role of miR-17 in renal fibrosis. miR-17 directly inhibited both H19 and fibronectin expression. H19 over-expression may become a sink for miR-17, thereby affecting the derepression of fibronectin. miR-17 is emerged as a regulator at the post-transcriptional level. H19 plays as a gene regulator at the transcriptional level. The regulatory network integrates the transcriptional and post-transcriptional regulatory network of renal fibrosis.

Fibronectin is an adhesive glycoprotein and a major ECM constituent [32]. Fibronectin plays important role in several biological processes, including cell adhesion, cell migration, and cell proliferation. Fibrosis is the excessive accumulation of ECM [33]. LncRNA-H19 functioned as a ceRNA to regulate fibronectin levels by sponging miR-17. Thus, H19 regulation could alter fibronectin level, which in turn affected renal fibrosis.

In conclusion, we showed that H19 level was significantly up-regulated in TGF-β2-induced HK-2 cell fibrosis *in vitro* and unilateral ureteral obstruction (UUO)-induced renal fibrosis *in vivo*. H19 knockdown attenuated renal fibrosis through lncRNA-H19/miR-17/fibronectin regulatory network. Antagonism of H19 may represent a novel anti-fibrotic treatment in renal diseases.

## MATERIALS AND METHODS

### Cell culture

HK-2 human proximal tubular epithelial cells were obtained from American Type Culture Collection (ATCC) and cultured in the medium (DMEM/F12, 1:1 mixture; Hyclone) containing 10% fetal bovine serum (FBS; Hyclone) in a regular CO_2_ incubator at 37°C under 5% CO_2_/95% air.

### UUO animal model

The mouse UUO model was induced in 4-month old male C57BL/6J mice. Ureteral obstruction was conducted by ligating left ureter using 3-0 silk via left lateral incision. Sham-operated C57BL/6J mice were taken as the controls. Obstructed renal (UUO) and sham-operated renal was harvested at each day. The experimental operation was approved by the Ethics Review Committee for Animal Experimentation of Shanghai Jiao Tong University.

### Immunohistochemistry

Renal tissues were fixed in 4% paraformaldehyde in phosphate-buffered saline (PBS) and embedded in paraffin. Immunohistochemistry was performed in paraffin sections (3 μm thick) using a microwave-based antigen retrieval technique. After immunostaining, the sections were counterstained with hematoxylin. Percentages of positive staining areas were determined by the Image-Pro plus software (Media Cybernetics, Bethesda, MD).

### Transwell assay

To detect cell migration, HK-2 cells were placed in the upper chamber of a Transwell (1 × 10^5^ cells per chamber, pore size 8 μm, Corning) coated with fibronectin (1 μg/ml). After the required treatment, HK-2 cells were allowed to migrate for 5 h at 37°C. Non-migrated cells were scraped off using a cotton swab. The migrated cells remaining on the bottom surface were counted after crystal violet staining, and observed using a microscope.

### RNA isolation and quantitative reverse transcriptase-polymerase chain reaction (RT-PCR)

Total RNAs were extracted from renal tissue or HK-2 cells using Trizol reagent (Invitrogen, Carlsbad, CA). Reverse transcription was performed using the SuperScript First-Strand Synthesis System (Invitrogen, Carlsbad, CA). All PCR experiments were performed using a QuantiTect SYBR Green PCR kit (Qiagen) on a PikoReal Real-Time PCR System (Thermo Scientific). For the quantitative PCR reactions, 30–50 μg cDNA was added to a 20 μl reaction mixture containing 10 μl of 2× Power SYBR Green PCR Master Mix and 0.4 μl of each primer (25 pmol). Comparative C_t_ method was used to detect target gene expression in the test samples relative to the control samples.

### *In vitro* gene transfection

HK-2 cells were seeded at 5 × 10^5^ cells per well in 6-well plates. The following day, medium was replaced with OptiMEM (Invitrogen). These cells were transfected with miRNA mimic at 100 nmol/L using Oligofectamine (Invitrogen). The negative mimic or siRNA was transfected at the same concentration. Cells were harvested two days 48 h after transfection.

### Enzyme-linked immunosorbent assay (ELISA)

MMP-2 and MMP-9 ELISA kit (R&D Systems) was used to detect the activity of MMP-2 and MMP-9 in cell culture supernatants according to the manufacturer's instruction.

### Western blot analysis

Protein from renal tissues and HK-2 cells were extracted using protein lysis buffer. About 50 μg protein was subjected to 10–12% SDS-PAGE and transferred onto PVDF membranes. Membranes were blocked in 5% BSA for 1 h at room temperature. All primary and secondary antibody incubations were last for 3 h at room temperature. Signal was detected by chemiluminescence. The images were captured on the XRS Chemidoc system (BioRad) and analyzed by Quantity One software (BioRad).

### Statistical analyses

All data was expressed as the mean ± SEM and analyzed using one-way ANOVA, followed by *t* test using SPSS 13.0 software (Chicago, IL).

## SUPPLEMENTARY MATERIALS AND FIGURES


