# Supplementary material for: Long non-coding RNA-H19 antagonism protects against renal fibrosis
Source: Oncotarget. 2016 Jul 6;7(32):51473–81. doi: 10.18632/oncotarget.10444 (PMC5239489; doi:10.18632/oncotarget.10444)
Supplement: Supplementary file 1 [file oncotarget-07-51473-s001.pdf]

Long non-coding RNA-H19 antagonism protects against renal fibrosis

Supplementary Materials

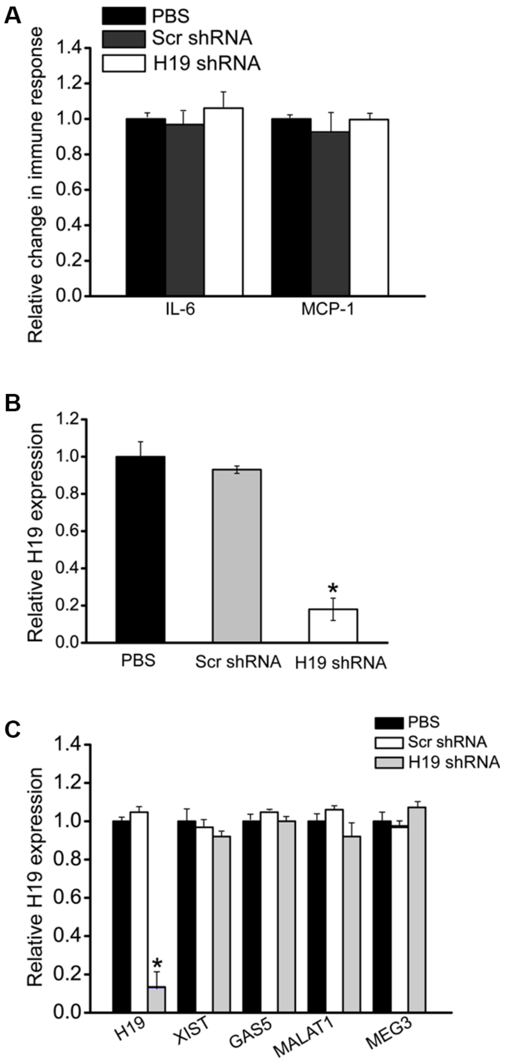

Supplementary Figure S1: H19 shRNA injection down-regulates H19 expression *in vivo*. (**A–C**) Four-month old C57BL/6 mice were injected subcutaneously with H19 shRNA lenti-virus. Scrambled shRNA lenti-virus or PBS was injected as the controls. Target sequence for mouse H19 shRNA design was 5′-GCGTAAGAGAATATATGAC -3′. ELISAs were conducted to the serum levels of IL-6 and MCP-1 ($n = 5$ animals per group, (A). qRT-PCRs were conducted to detect renal H19 levels ($n = 5$ animals per group, (B). qRT-PCRs were conducted to detect renal expression levels of long non-coding RNAs, including MALAT1, MALAT1, GAS5, MEG3, and H19 ($n = 5$ animals per group, (C). The data was shown as fold increase compared with PBS-injected group. $*P < 0.05$ versus PBS-injected group. All data were from three independent experiments.

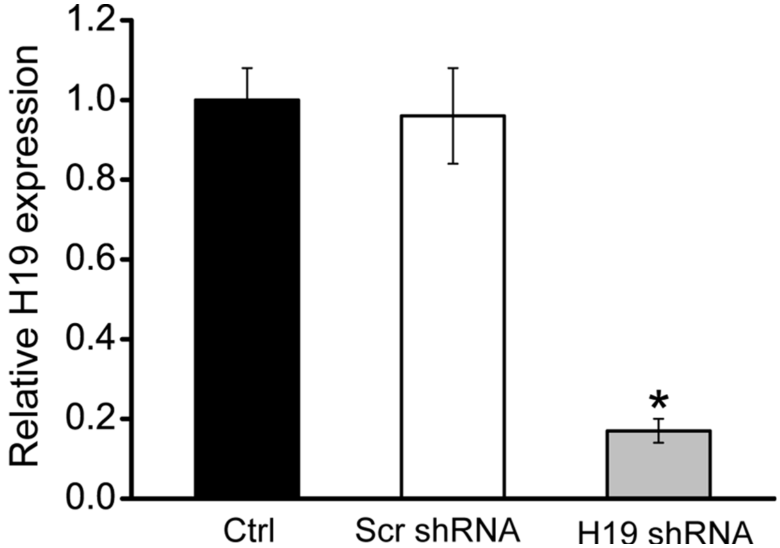

Supplementary Figure S2: H19 siRNA injection down-regulates H19 expression *in vitro*. HK-2 cells were transfected with H19 siRNA, scrambled siRNA (Scr), or left untreated (Ctrl) for 48 h. Target sequence for human H19 siRNA design was 5′- GCCTCCACGACTCTGTTTC-3′. qRT-PCRs were conducted to detect H19 expression. The data was shown as fold increase compared with Ctrl group (n = 4;*P < 0.05 versus Ctrl group). All data were from three independent experiments.
